# Supplementary material for: Antibody targeting intracellular oncogenic Ras mutants exerts anti-tumour effects after systemic administration
Source: Nat Commun. 2017 May 10;8:15090. doi: 10.1038/ncomms15090 (PMC5436137; doi:10.1038/ncomms15090)
Supplement: Supplementary Information — Supplementary Figures, Supplementary Tables and Supplementary References [file ncomms15090-s1.pdf]

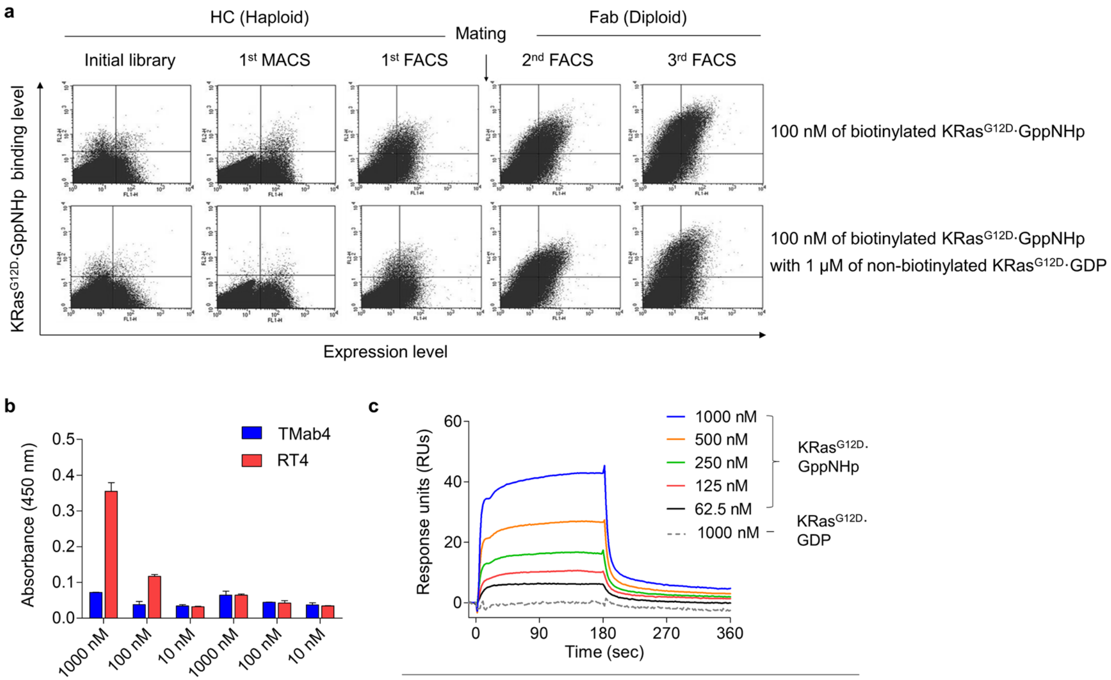

	k_a ($M^{-1}s^{-1}$)	k_d (s^{-1})	K_D (M)
RT4	2.99×10^4	3.5×10^{-3}	1.1×10^{-7}

Supplementary Figure 1 Isolation and characterization of GppNHp-bound active KRas^{G12D}-specific binding RT4. (**a**) Flow cytometric analysis of antigen binding and expression levels of HC in the haploid library and Fab in the mated diploid yeast library. (**b**) Binding activity of RT4 and TMab4 with KRas^{G12D}·GppNHp and KRas^{G12D}·GDP, determined by direct ELISA. ELISA plates were coated with 5 μg ml^{-1} of antibodies and then incubated with the antigen at concentrations of 10, 100, and 1000 nM. Error bars represent the mean ± s.d. (n = 3). (**c**) Representative SPR sensorgrams showing the kinetic interactions of RT4 with KRas^{G12D}·GppNHp. The injection of KRas^{G12D}·GDP (1 μM) (dashed line) exhibited negligible binding to RT4. The inset table shows the kinetic interaction parameters.

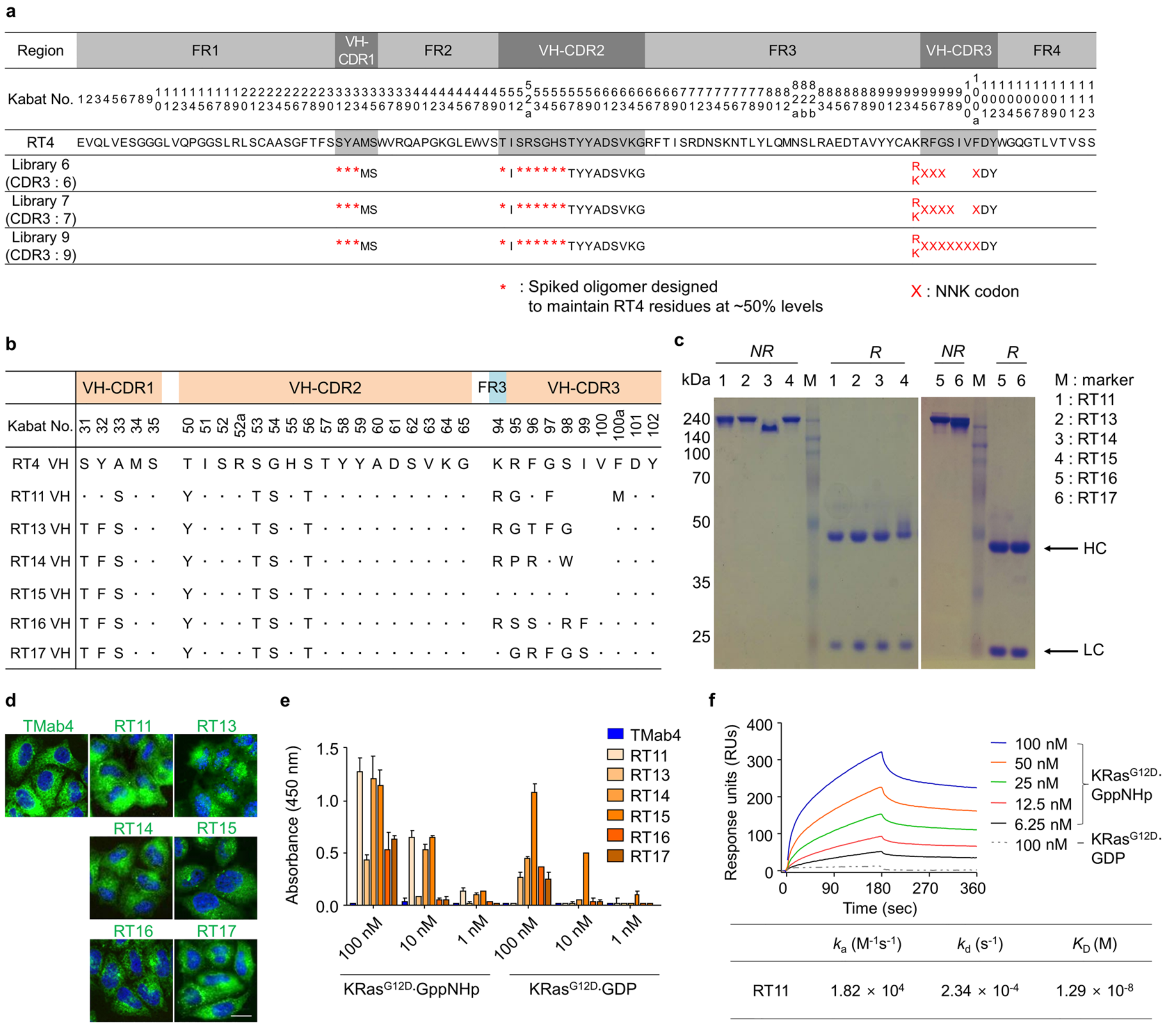
a
Region
FR1
VH-CDR1
FR2
VH-CDR2
FR3
VH-CDR3
FR4
Kabat No.
RT4
EVQLVESGGGLVQPGGSLRLSCAASGFTFSSYAMSWVRQAPGKGLEWSTISRSGHSTYYADSVKGRFTISRDNSKNTLYLQMNSLRAEDTAVYYCAKRFGSIVFDYWGQGTLVTVSS
Library 6 (CDR3 : 6)
Library 7 (CDR3 : 7)
Library 9 (CDR3 : 9)
* : Spiked oligomer designed to maintain RT4 residues at ~50% levels
X : NNK codon
b
VH-CDR1
VH-CDR2
FR3
VH-CDR3
Kabat No.
RT4 VH
RT11 VH
RT13 VH
RT14 VH
RT15 VH
RT16 VH
RT17 VH
c
NR
R
kDa
M : marker
1 : RT11
2 : RT13
3 : RT14
4 : RT15
5 : RT16
6 : RT17
HC
LC
d
TMab4
RT11
RT13
RT14
RT15
RT16
RT17
e
Absorbance (450 nm)
100 nM
10 nM
1 nM
KRasG12D·GppNHp
KRasG12D·GDP
f
Response units (RUs)
Time (sec)
100 nM
50 nM
25 nM
12.5 nM
6.25 nM
KRasG12D·GppNHp
KRasG12D·GDP
k_a (M-1s-1)
k_d (s-1)
K_D (M)
RT11
1.82 × 10^4
2.34 × 10^-4
1.29 × 10^-8

Supplementary Figure 2 Affinity maturation of RT4 and biochemical characterization of the isolated RT4 variants. (**a**) Library construction scheme. Amino acid sequence alignment of RT4 VH showing the positions of conserved and mutated residues in the three libraries differing in the length of VH-CDR3, dubbed library 6, 7, and 9. The residues (31–33 residues) of VH-CDR1 and (50, 52–56 residues) of VH-CDR2, highlighted by asterisks (*), were randomly mutated, while maintaining the original amino acids at each residue of RT4 at a frequency of ~50%, using designed oligonucleotides. The VH-CDR3 region (residues 95–100a) was randomized with the degenerated codon of NNK, as indicated by 'X', which resulted in variation in the total length of 6, 7, and 9 residues. The framework residue of 94 was also permutated with degenerate codons of ARG to introduce Arg or Lys, in an attempt to cover the most conserved amino acids at the residues in human antibodies. Numbering is according to the Kabat definition. (**b**) Amino acid sequence alignment of the isolated RT4 variants. (**c**) Reducing ('*R*') and non-reducing ('*NR*') SDS-PAGE analyses of the purified RT4 variants (each 5 μg) in the human IgG1 format. (**d**) Cellular internalization and cytosolic localization of the purified TMab4 (green) and RT4 variants (green) in HeLa cells treated with antibodies (1 μM) for 6 h at 37 °C and then analyzed by confocal microscopy. Nuclei were counterstained with Hoechst 33342 (blue). Scale bar, 20 μm. (**e**) Binding specificity of RT4 variants to $KRas^{G12D}$·GppNHp or $KRas^{G12D}$·GDP, determined by direct ELISA. ELISA plates were coated with 5 μg ml^{-1} of antibodies and then incubated with the antigen at concentrations of 1, 10, and 100 nM. Error bars represent the mean ± s.d. (n = 3). (**f**) Representative SPR sensorgrams showing the kinetic interactions of RT11 with $KRas^{G12D}$·GppNHp. The injection of $KRas^{G12D}$·GDP (1 μM) (dashed line) exhibited negligible binding to RT11. The inset table shows the kinetic interaction parameters.

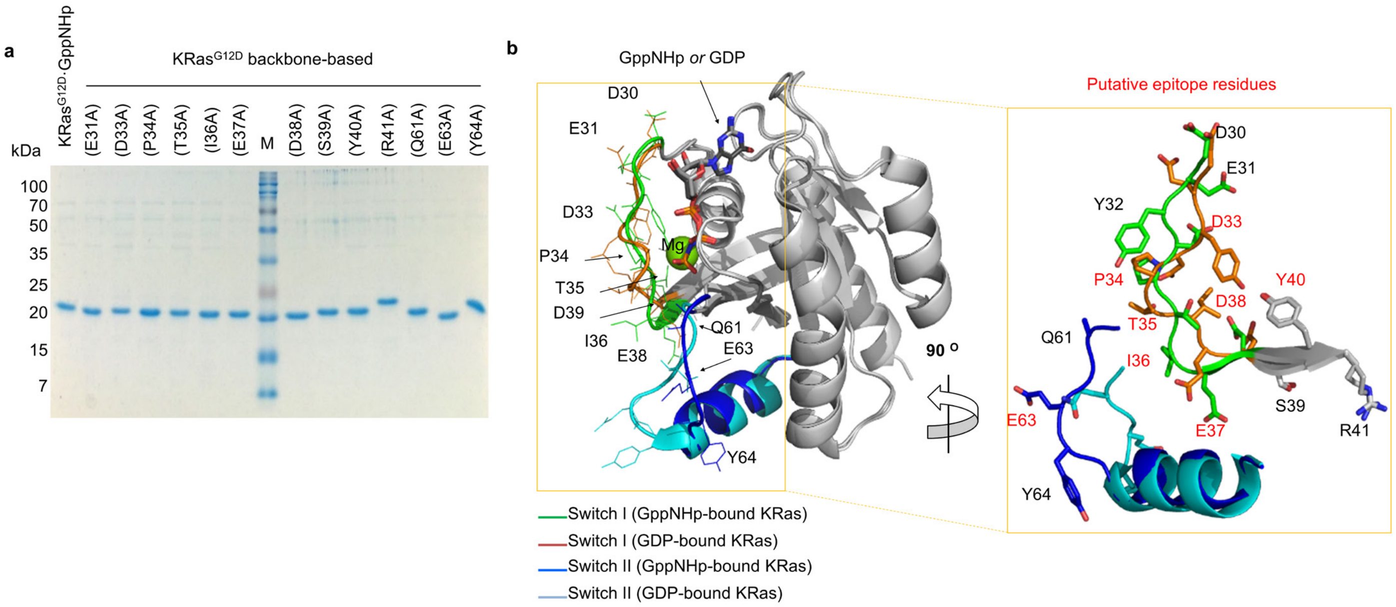

Supplementary Figure 3 Epitope mapping of RT11 iMab by alanine-scanning mutagenesis. (**a**) Reducing SDS-PAGE analyses of bacterially expressed and purified KRas^{G12D} mutants (residue 1–168) with Ala mutation at the indicated residues. (**b**) Highlight of the epitopes of RT11 (red in the right panel) on the three-dimensional structure of KRas, determined by alanine scanning mutagenesis as shown in Fig. 1e. Three-dimensional structural comparison between GDP-bound inactive KRas (PDB ID: 4EPV)[1] and GppNHp-bound active KRas (PDB ID: 3GFT) shows conformational differences only in the switch I and II regions. Both GppNHp and GDP are shown as sticks colored according to heteroatoms (oxygen: red; nitrogen: blue; carbon: gray; phosphorus: orange), and the Mg^{2+} ion is shown as a green sphere. The switch I and switch II regions of KRas·GppNHp and KRas·GDP are represented by green/blue or orange/cyan wires, respectively, with Ala mutation residues indicated. The rest of the protein structure is displayed in gray ribbon. The images were generated using PyMol program (Schrödinger).

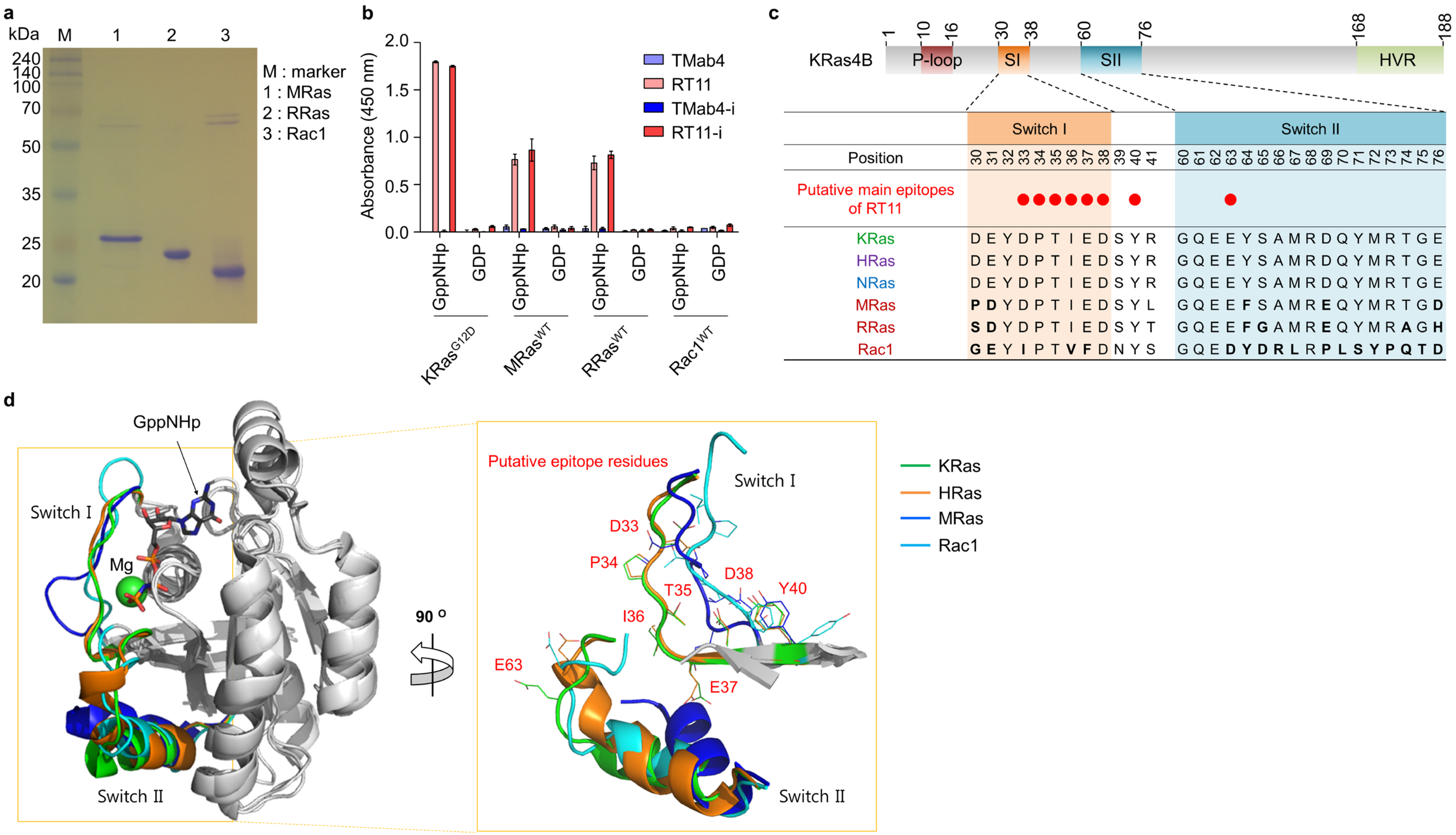
a
kDa
M
1
2
3
240
140
100
70
50
35
25
20
M : marker
1 : MRas
2 : RRas
3 : Rac1
b
Absorbance (450 nm)
2.0
1.5
1.0
0.5
0.0
TMab4
RT11
TMab4-i
RT11-i
GppNHp
GDP
KRas^{G12D}
MRas^{WT}
RRas^{WT}
Rac1^{WT}
c
KRas4B
1
10
16
30
38
60
76
168
188
P-loop
SI
SII
HVR
Switch I
Switch II
Position
Putative main epitopes of RT11
KRas D E Y D P T I E D S Y R G Q E E Y S A M R D Q Y M R T G E
HRas D E Y D P T I E D S Y R G Q E E Y S A M R D Q Y M R T G E
NRas D E Y D P T I E D S Y R G Q E E Y S A M R D Q Y M R T G E
MRas P D Y D P T I E D S Y L G Q E E F S A M R E Q Y M R T G D
RRas S D Y D P T I E D S Y T G Q E E F G A M R E Q Y M R A G H
Rac1 G E Y I P T V F D N Y S G Q E D Y D R L R P L S Y P Q T D
d
GppNHp
Switch I
Mg
Switch II
90 °
Putative epitope residues
Switch I
D33
P34
T35
I36
D38
Y40
E63
E37
Switch II
KRas
HRas
MRas
Rac1

Supplementary Figure 4 RT11/RT11-i binds weakly to the active forms of MRas and RRas, but does not cross-react with Rac1. (**a**) Reducing SDS-PAGE analyses of bacterially expressed and purified $MRas^{WT}$, $RRas^{WT}$, and $Rac1^{WT}$. (**b**) Binding activity of the indicated antibodies to the GppNHp-bound active forms or GDP-bound inactive forms of $MRas^{WT}$, $RRas^{WT}$, and $Rac1^{WT}$, compared to that of active $KRas^{G12D}$. ELISA plates were coated with 5 μg ml^{-1} of antibodies and then incubated with the antigen at 100 nM. Error bars represent the mean ± s.d. (n = 3). (**c**) Comparison of KRas residues in the switch I and II regions with those of HRas and NRas as well as other Ras superfamily GTPases such as MRas, RRas, and Rac1. The residues identified as putative binding epitopes of RT11 on $KRas^{G12D}$ by alanine scanning mutagenesis are highlighted as red circles. The residue numbering is based on the sequence of KRas. (**d**) Comparison of the crystal structures of GppNHp-bound active KRas (PDB ID: 3GFT), HRas (PDB ID: 1CTQ)[2], MRas (PDB ID: 1X1S)[3], and Rac1 (PDB ID: 5FI0)[4], highlighting the putative binding epitopes of RT11 on KRas (right panel). The switch I and II regions of KRas superimpose well with HRas showing root-mean-square-deviations of 0.45Å, but poorly with MRas and Rac1 showing root-mean-square-deviations of 2.06 and 2.47 Å, respectively. The crystal structure of GppNHp-bound RRas has not yet been determined, but it is expected to be similar to that of MRas rather than to that of KRas based on primary sequence homology (60.9% and 51.8% sequence identity with MRas and KRas, respectively)[5,6]. The images were generated using the PyMol program (Schrödinger).

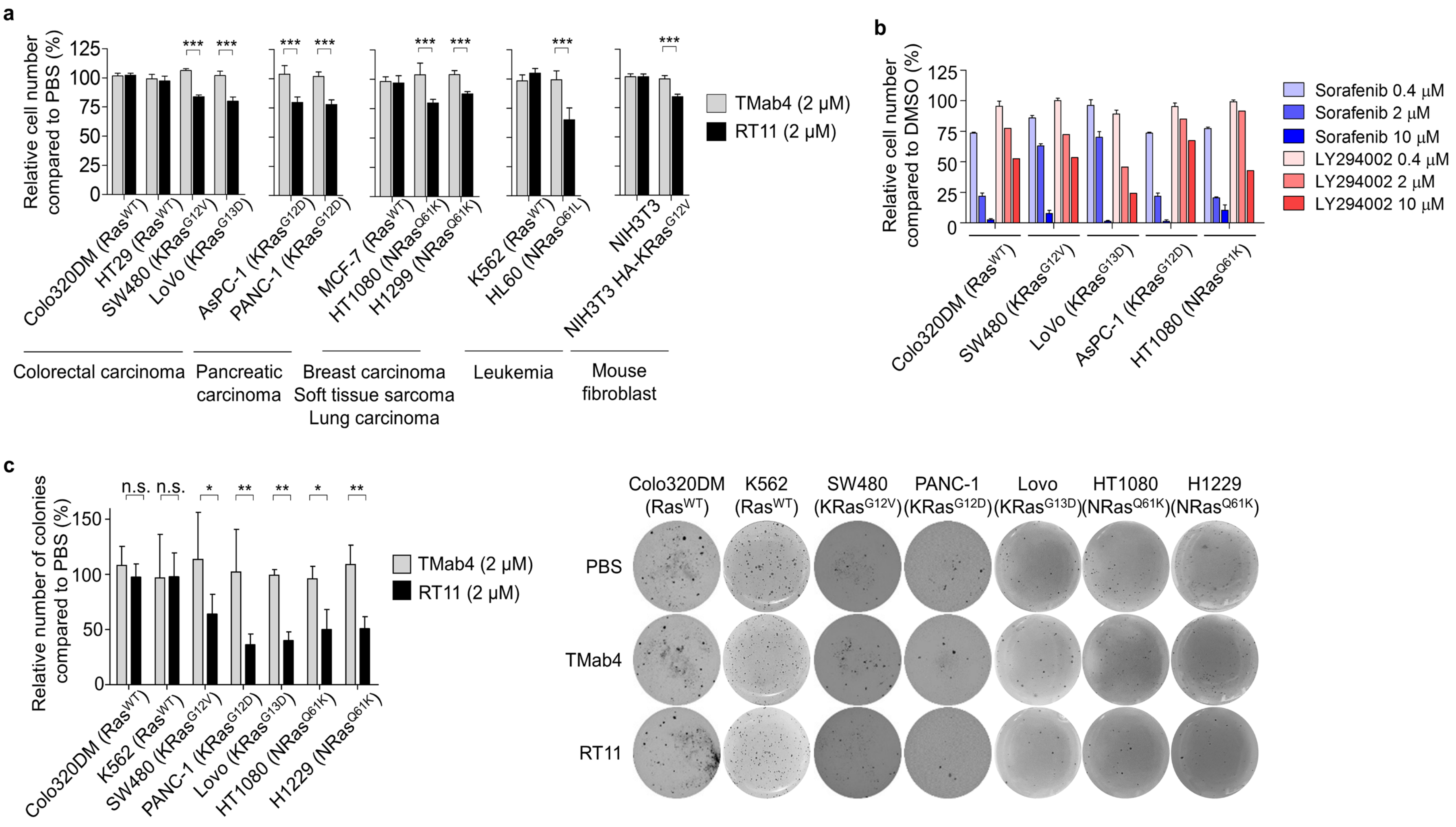
a
Relative cell number compared to PBS (%)
TMab4 (2 μM)
RT11 (2 μM)
Colo320DM (RasWT)
HT29 (RasWT)
SW480 (KRasG12V)
LoVo (KRasG13D)
AsPC-1 (KRasG12D)
PANC-1 (KRasG12D)
MCF-7 (RasWT)
HT1080 (NRasQ61K)
H1299 (NRasQ61K)
K562 (RasWT)
HL60 (NRasQ61L)
NIH3T3
NIH3T3 HA-KRasG12V
Colorectal carcinoma
Pancreatic carcinoma
Breast carcinoma
Soft tissue sarcoma
Lung carcinoma
Leukemia
Mouse fibroblast
b
Relative cell number compared to DMSO (%)
Sorafenib 0.4 μM
Sorafenib 2 μM
Sorafenib 10 μM
LY294002 0.4 μM
LY294002 2 μM
LY294002 10 μM
Colo320DM (RasWT)
SW480 (KRasG12V)
LoVo (KRasG13D)
AsPC-1 (KRasG12D)
HT1080 (NRasQ61K)
c
Relative number of colonies compared to PBS (%)
n.s. n.s.
TMab4 (2 μM)
RT11 (2 μM)
Colo320DM (RasWT)
K562 (RasWT)
SW480 (KRasG12V)
PANC-1 (KRasG12D)
Lovo (KRasG13D)
HT1080 (NRasQ61K)
H1229 (NRasQ61K)
PBS
TMab4
RT11

Supplementary Figure 5 RT11 inhibits the growth of tumor cells harboring oncogenic Ras mutants, but not those with Ras^{WT}. (**a, b**) Cellular proliferation assay in indicated cells, treated twice at 0 and 72 h with the indicated concentration of antibody (RT11 or TMab4) (**a**) or the pharmacological inhibitor (Raf kinase inhibitor sorafenib or PI3K-Akt inhibitor LY294002) (**b**) for 6 d. Error bars represent the mean ± s.d. (n = 3). $^{**}P < 0.01$, $^{***}P < 0.001$. (**c**) Inhibition of soft agar colony formation of tumor cells by RT11. Anchorage-independent cell growth was examined by soft agar colony formation assays using the oncogenic KRas mutant, NRas mutant, and Ras^{WT} cells, treated with RT11 (2 μM) and TMab4 (2 μM) every 72 h for 2–3 weeks. Following treatment, the number of colonies (diameter > 200 μm) was counted after BCIP/NBT staining, as shown in the pictures of the representative soft agar plates (right). The results are presented as percentages compared to the PBS-treated control. Error bars represent the mean ± s.d. (n = 3). $^{*}P < 0.05$, $^{**}P < 0.01$; n.s., not significant. The pictures are representative of three independent experiments.

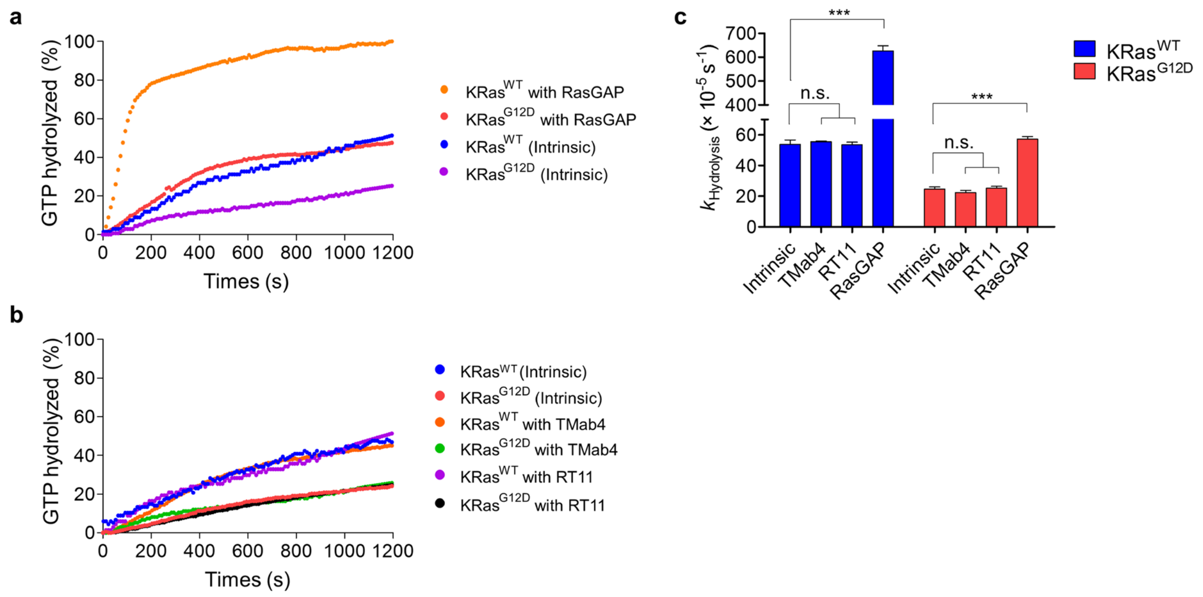

Supplementary Figure 6 RT11 does not affect the intrinsic GTPase activity of KRas^{WT} and KRas^{G12D} proteins. (**a**,**b**) Kinetic profiles of GTP hydrolysis of GTP-loaded KRas^{WT} and KRas^{G12D} proteins in the absence (intrinsic GTPase activity) and presence of GTPase-activating RasGAP protein (GAP-stimulated GTPase activity) (**a**) and the indicated antibodies (**b**). In (**a**), GAP-stimulated GTP hydrolysis was accessed with the catalytic domain of RasGAP (residues 714–1047)[7]. (**c**) Comparisons of GTP hydrolysis rate constants (*k*). GTP hydrolysis was determined by continuously measuring the release of phosphate using a purine nucleoside phosphorylase–based colorimetric assay[7]. The concentration of phosphate released *vs*. time was plotted and the first-order rate constant was determined. In (**c**), error bars represent the mean ± s.d. of three independent experiments. $***P < 0.001$; n.s., not significant. The intrinsic and RasGAP-stimulated GTP hydrolysis rate constants of KRas^{WT} and KRas^{G12D} were comparable to those reported earlier[7].

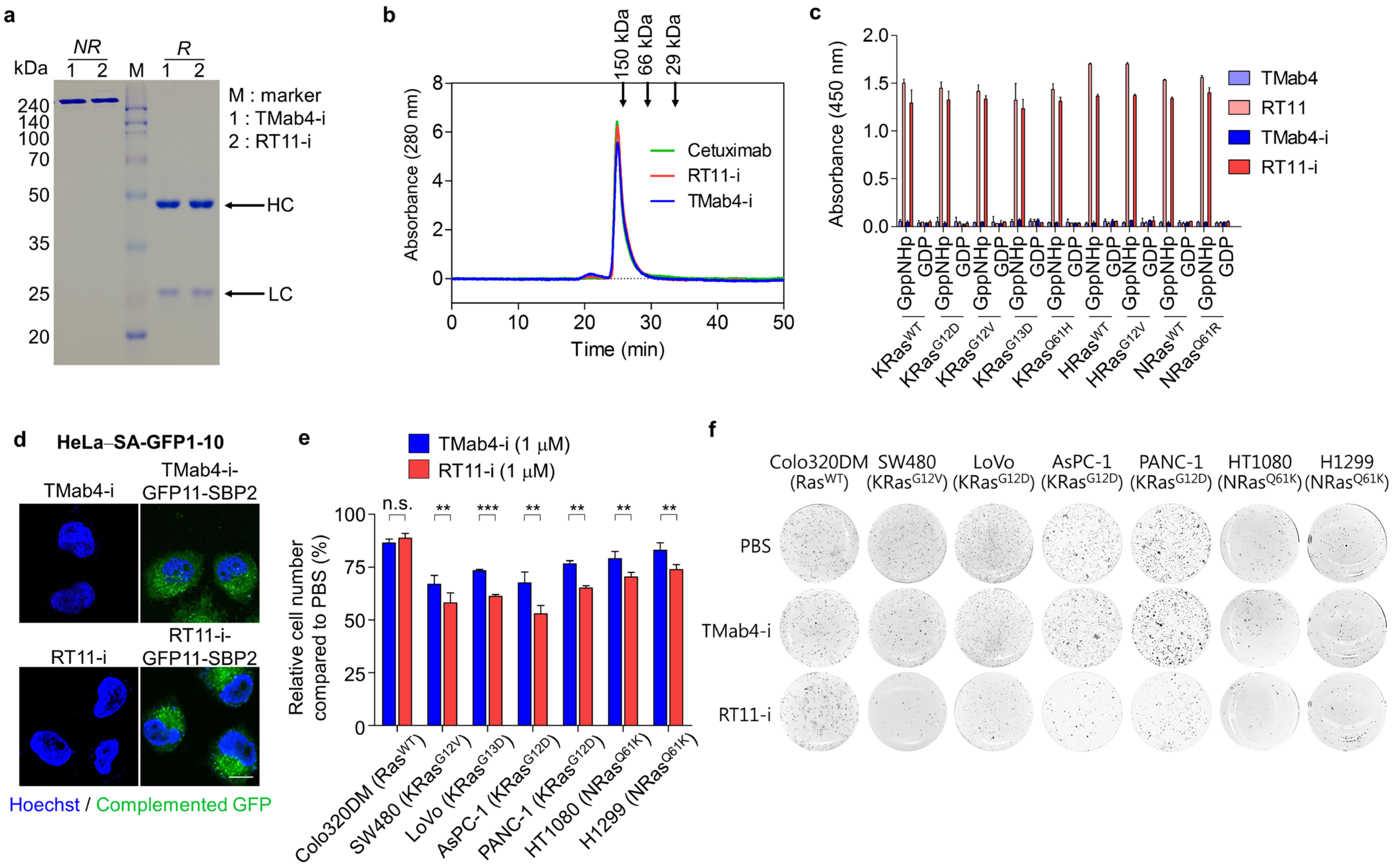

Supplementary Figure 7 Biochemical and biological characterization of RGD10 peptide-fused RT11-i iMab. (**a**) Reducing ('*R*') and non-reducing ('*NR*') SDS-PAGE analyses of the purified antibodies (each 5 μg) in the human IgG1 format. (**b**) Size exclusion elution profiles of purified TMab4-i and RT11-i (50 μl of 100 μg ml^{-1}) monitored at 280 nm. Cetuximab (50 μl of 100 μg ml^{-1}) was included as a control. The

arrows indicate the elution positions of molecular weight standards. Two independent analyses were performed with the same results. (**c**) ELISA showing the selective binding of RT11-i, but not TMab4-i, to GppNHp-bound active Ras variants over GDP-bound inactive forms at comparable levels to those observed with RT11. Error bars represent the mean ± s.d. (n = 3). (**d**) Cellular internalization and cytosol localization of TMab4-i and RT11-i, assessed by confocal microscopy for complemented GFP signals (green) in HeLa-SA-GFP1-10 cells after treatment with 1 μM of TMab4-i-GFP11-SBP2 and RT11-i-GFP11-SBP2 for 6 h. Scale bar, 20 μm. Nuclei were counterstained with Hoechst 33342 (blue). Scale bar, 20 μm. (**e**) Cellular proliferation assay under monolayer culture conditions with the indicated cells, treated twice at 0 and 72 h with 1 μM of RT11-i or TMab4-i for 6 d. Error bars represent the mean ± s.d. (n = 3). **P < 0.01, ***P < 0.001; n.s., not significant. (**f**) Inhibition of soft agar colony formation by RT11-i compared to that with TMab4-i, as described in Fig. 4f. The pictures are representative of three independent experiments.

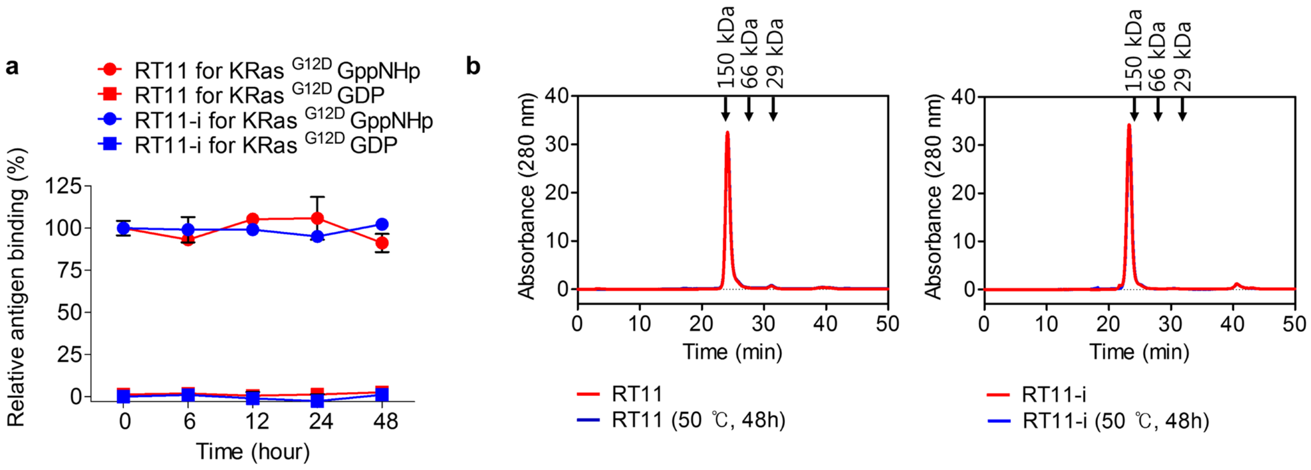

Supplementary Figure 8 RT11-i possesses comparable thermal stability to that of RT11. (**a**,**b**) Thermal stability of RT11 and RT11-i was assessed by ELISA for the selective antigen binding (**a**) and size exclusion chromatography (**b**) after incubating the antibodies (2 mg ml^{-1}) at 50 °C for the indicated periods. In (**a**), the binding activity (%) was relative to the initial binding of antibody stored at 4 °C. Error bars represent the mean ± s.d. (n = 3). In (**b**), size exclusion chromatogram of antibodies (20 µl of 1 mg ml^{-1}) was monitored at 280 nm. Antibody stored at 4 °C was included as a control. Two independent analyses were performed with the same results. The arrows indicate the elution positions of molecular weight standards. SEC analysis of purified antibodies was performed on the Agilent 1100 high performance liquid chromatography system using a superdex™200 10/300GC (10 mm × 300 mm, GE Healthcare) size-exclusion column with a mobile phase of PBS buffer (pH 7.4) at a flow rate 0.5 ml min^{-1}.

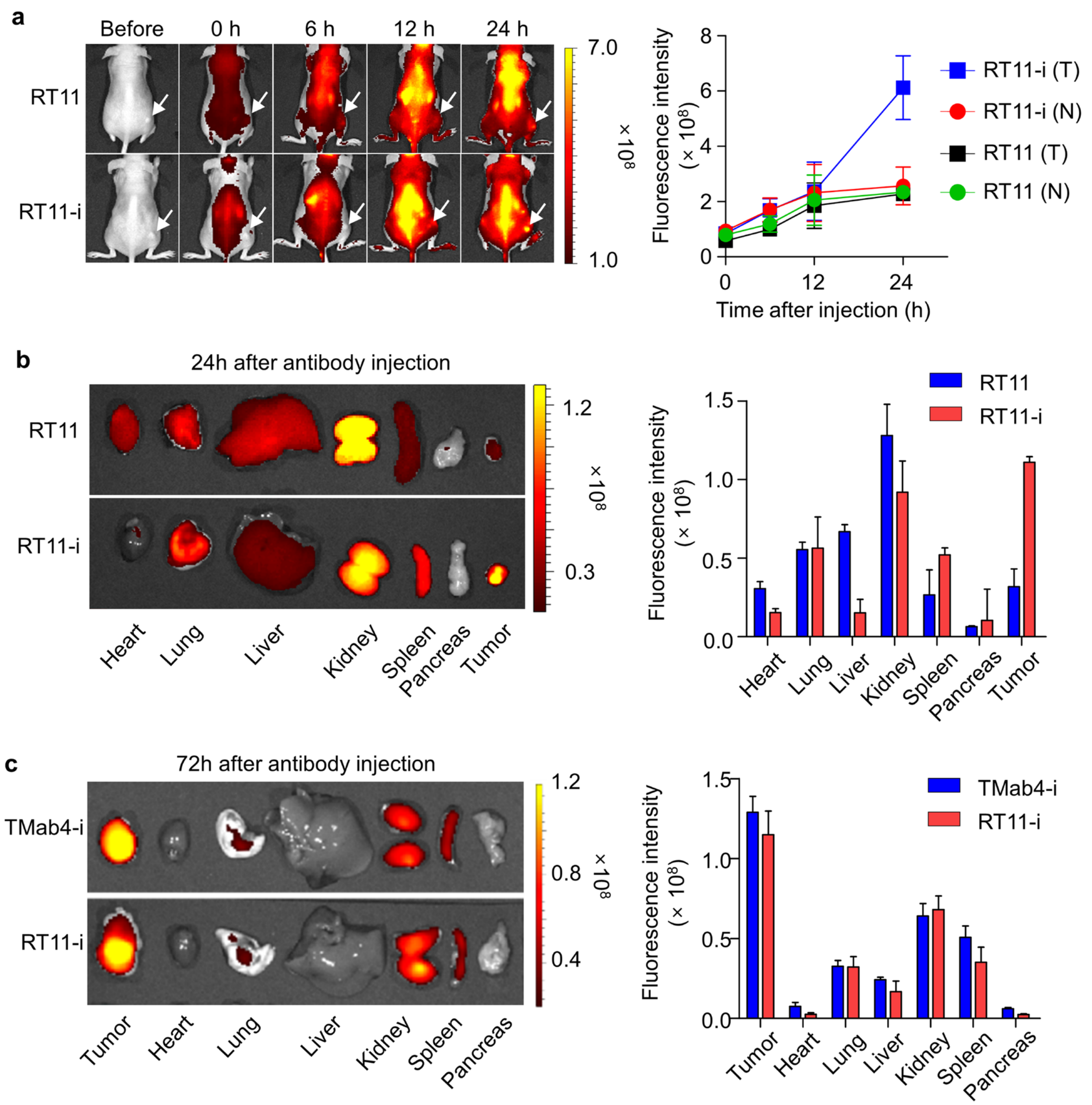
a
Before
0 h
6 h
12 h
24 h
RT11
RT11-i
7.0
1.0
×10^8
Fluorescence intensity (× 10^8)
Time after injection (h)
RT11-i (T)
RT11-i (N)
RT11 (T)
RT11 (N)
b
24h after antibody injection
RT11
RT11-i
1.2
0.3
Heart
Lung
Liver
Kidney
Spleen
Pancreas
Tumor
c
72h after antibody injection
TMab4-i
RT11-i
1.2
0.8
0.4
Tumor
Heart
Lung
Liver
Kidney
Spleen
Pancreas

Supplementary Figure 9 Preferential tumor tissue accumulation of TMab4-i and RT11-i in SW480 tumor-bearing xenograft mice. (a,b) Comparison of biodistribution between RT11 and RT11-i, evaluated by intravenously injecting Dylight755-labeled antibodies (20 μg per mouse) into SW480 xenograft tumor-bearing mice. In (**a**), representative whole body fluorescence images, which were acquired at the indicated times post-injection. Fluorescence intensities in the tumor tissue (T), as indicated by arrows, and normal tissues (N) were quantified. In (**b**), *ex vivo* analysis of fluorescence intensities of dissected tumors and normal organs, which were acquired at 24 h post-injection. The right panel shows quantified fluorescence intensities of each tissue or organ. In (**a,b**), error bars represent the mean ± s.d. (n = 6 per group). **(c)** *Ex vivo* analysis of fluorescence intensities for isolated tumors and normal organs 72 h after intravenous injection of Dylight755-labeled TMab4-i and RT11-i antibodies (20 μg per mouse) into mice bearing SW480 tumor xenografts, as shown in Fig. 5b. Error bars represent the mean ± s.d. (n = 5 per group). In (**b,c**), tumor tissue and normal organs of one representative mouse from each group are shown. In (**a-c**), fluorescence intensities were quantified as radiant efficiency (photons s^{-1} cm^{-2} $steradian^{-1}$ per μW cm^{-2}) using Living Image software.

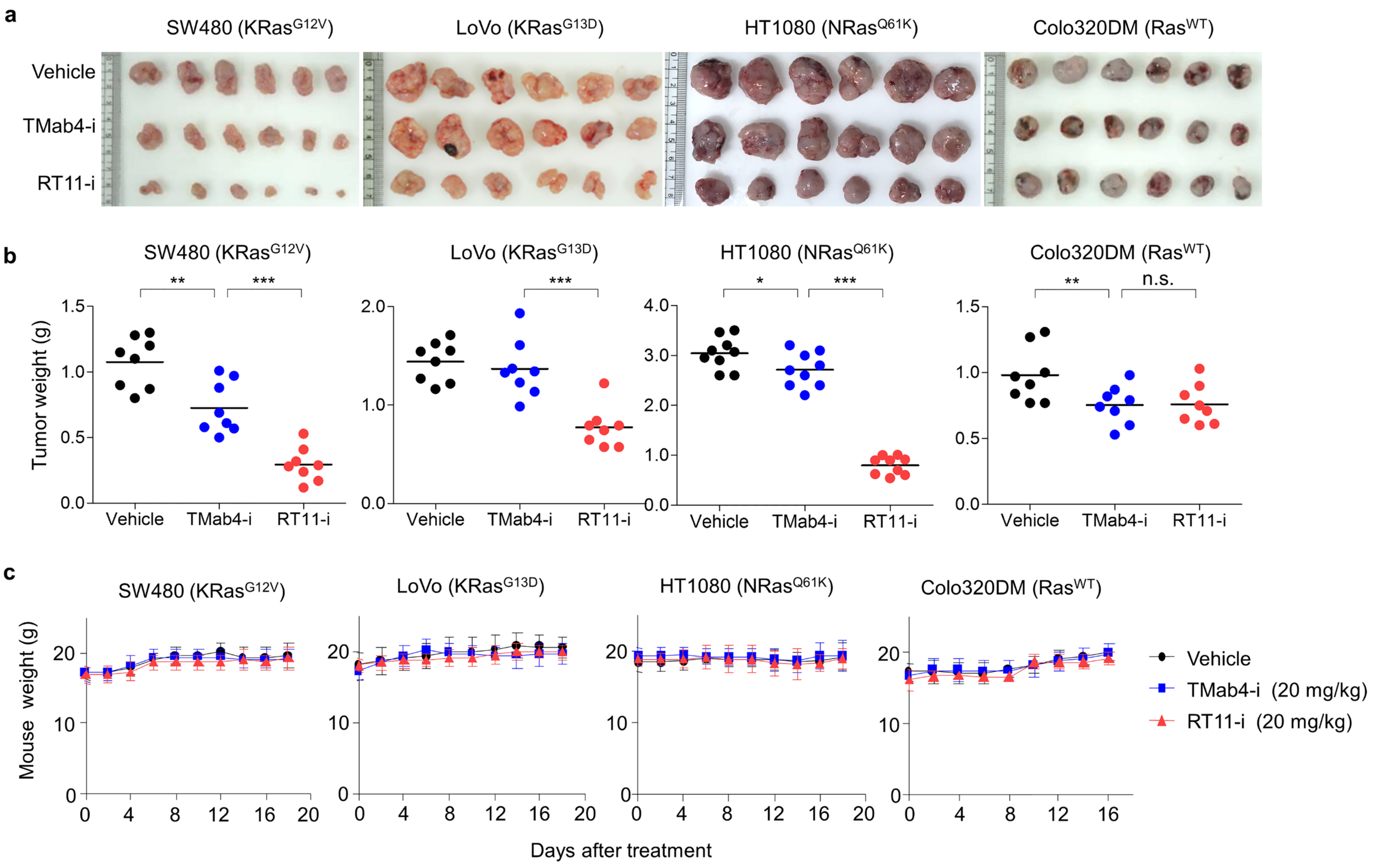
a
SW480 (KRasG12V)
LoVo (KRasG13D)
HT1080 (NRasQ61K)
Colo320DM (RasWT)
Vehicle
TMab4-i
RT11-i
b
Tumor weight (g)
**
***
*
n.s.
c
Mouse weight (g)
Days after treatment
Vehicle
TMab4-i (20 mg/kg)
RT11-i (20 mg/kg)

Supplementary Figure 10 RT11-i suppresses the *in vivo* growth of oncogenic Ras mutant tumor xenografts in mice. (**a**) Representative photographs of tumors excised from mice following treatment described in Fig. 6a. (**b**) The weight of individual tumors from each treatment group, as described in Fig. 6a. *$P < 0.05$, **$P < 0.01$, ***$P < 0.001$; n.s., not significant. (**c**) Mouse weight measured during the treatments described in Fig. 6a. Notably no signs of systemic toxicity (e.g., no significant changes in body weight, gross appearance, or behavior) were observed in mice treated with TMab4-i or RT11-i, compared to those in vehicle-treated mice. In (**b,c**), error bars represent the mean ± s.d. (n = 8 per group).

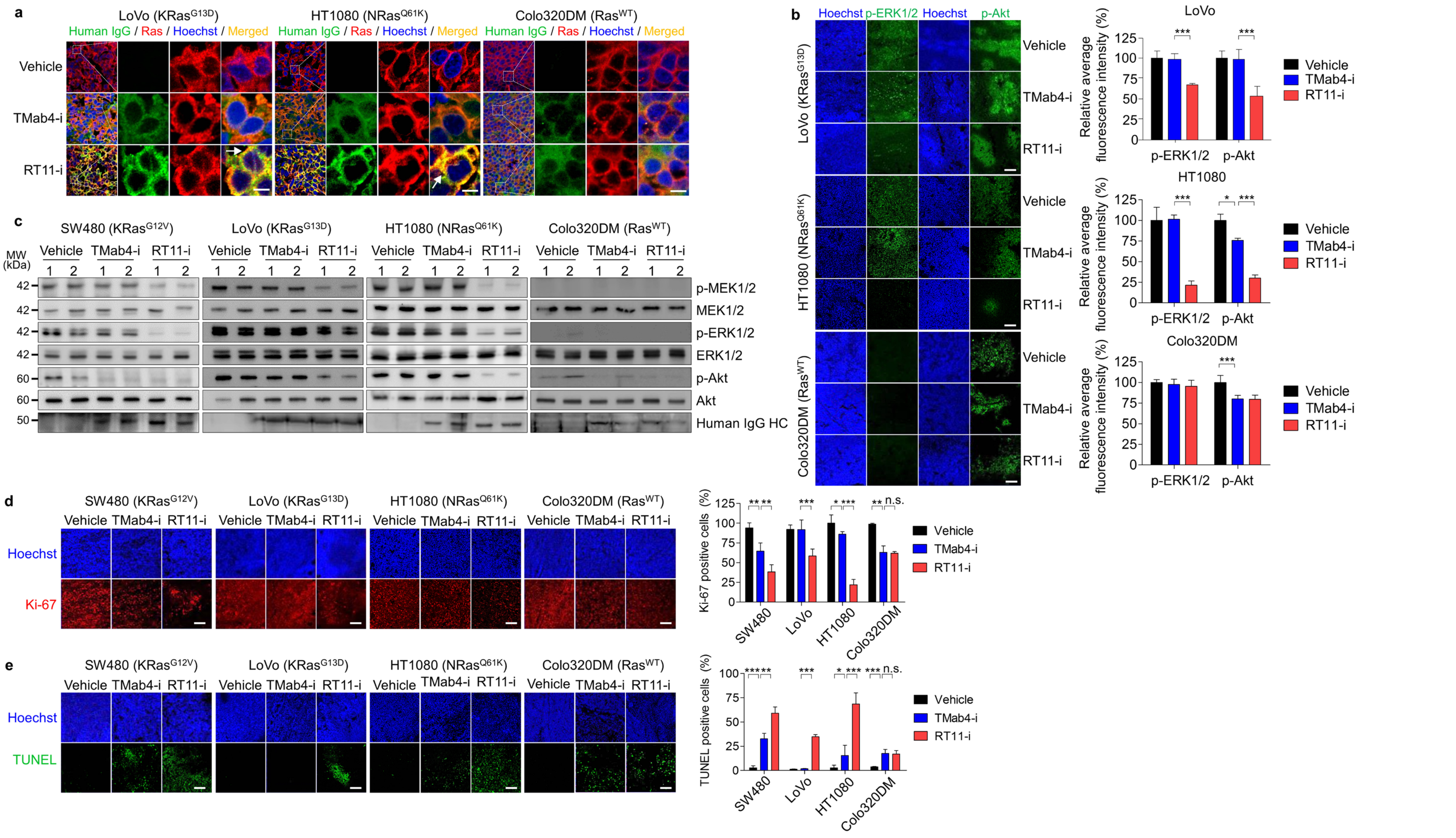
a
LoVo (KRas^{G13D})
HT1080 (NRas^{Q61K})
Colo320DM (Ras^{WT})
Human IgG / Ras / Hoechst / Merged
Vehicle
TMab4-i
RT11-i
b
Hoechst
p-ERK1/2
p-Akt
LoVo (KRas^{G13D})
HT1080 (NRas^{Q61K})
Colo320DM (Ras^{WT})
LoVo
HT1080
Colo320DM
Relative average fluorescence intensity (%)
p-ERK1/2
p-Akt
Vehicle
TMab4-i
RT11-i
c
SW480 (KRas^{G12V})
MW (kDa)
p-MEK1/2
MEK1/2
p-ERK1/2
ERK1/2
p-Akt
Akt
Human IgG HC
d
Hoechst
Ki-67
Ki-67 positive cells (%)
SW480
LoVo
HT1080
Colo320DM
n.s.
e
TUNEL
TUNEL positive cells (%)

Supplementary Figure 11 Immunohistochemical and western blot analyses of tumor tissues excised from mice, as described in Fig 6a. (**a**) Cellular penetration and co-localization of RT11-i (green), but not TMab4-i (green), with activated Ras (red) in oncogenic KRas mutant-harboring LoVo and NRas mutant-harboring HT1080 tumor tissues. In the Ras^{WT}-harboring Colo320DM tissues, both RT11-i (green) and TMab4-i (green) were internalized into cells, but did not significantly co-localize with Ras^{WT} proteins, likely because the Ras^{WT} proteins are not highly activated. Scale bar, 10 μm. (**b**) Analysis of p-ERK1/2 (green) and p-Akt (green) levels in LoVo, HT1080, and Colo320DM tumor tissues. Scale bar, 100 μm. The right panels show the percentage of relative fluorescence intensity compared to that in the vehicle-treated control. Error bars represent the mean ± s.d. of five random fields for each immunofluorescence sample (n = two tumors per group). (**c**) Western blot analysis of tumor tissue lysates prepared after treatment, as described in Fig. 6a to analyze the downstream signaling activation of Raf-MEK1/2-ERK1/2 and PI3K-Akt pathways. As indicated, two independent tumor tissue samples (sample 1 and sample 2) were analyzed. (**d,e**) Tumor cell proliferation and apoptosis were determined in tumor tissues through Ki-67 staining (**d**) and TUNEL staining (**e**), respectively. Scale bar, 100 μm. The right panels show the percentage of Ki-67-positive (**d**) and TUNEL-positive (**e**) cells compared to the number of Hoechst 33342-stained cells for each sample. Error bars represent the mean ± s.d. of five random fields for each immunofluorescence sample (n = two tumors per group). In (**a, b, d** and **e**), nuclei were counterstained with Hoechst 33342 (blue). In (**b,d** and **e**), statistical analyses were performed using a one-way ANOVA followed by the Newman-Keuls post-test. *$P < 0.05$, **$P < 0.01$, ***$P < 0.001$ *vs*. TMab4-i; n.s., not significant. Images are representative of at least two independent experiments.

Figure 2c

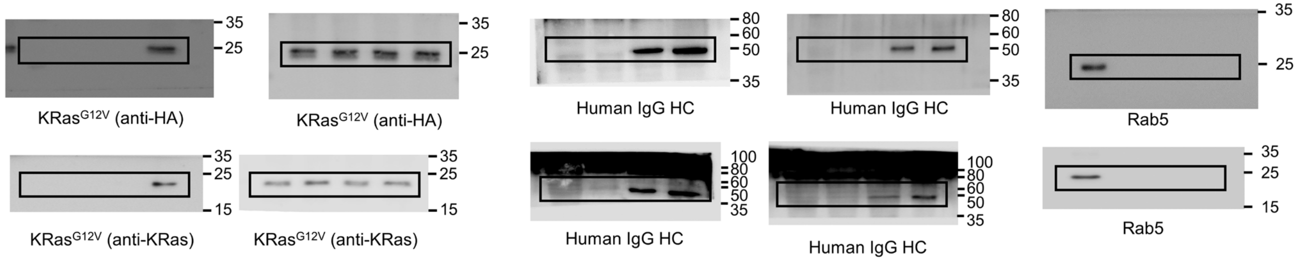

Figure 3c

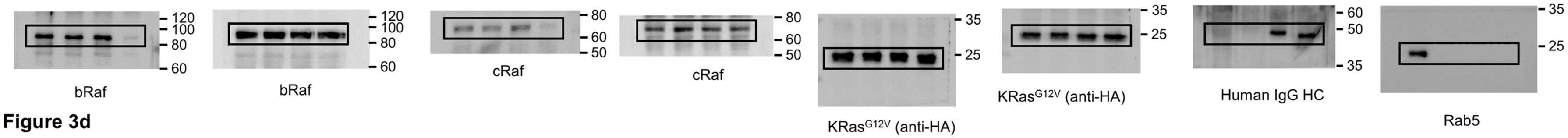

Figure 3d

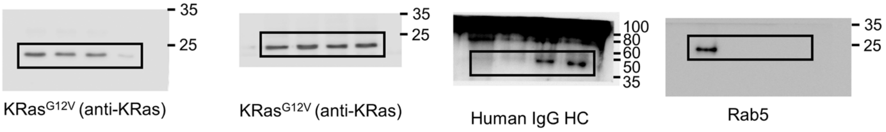

Figure 3e

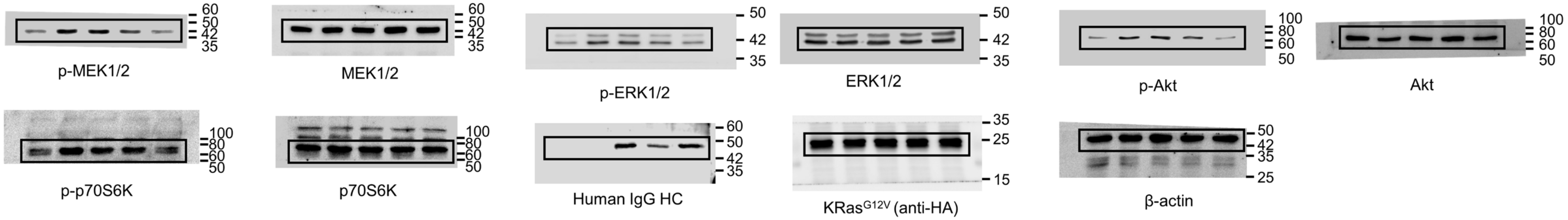

Supplementary Figure 12 Unprocessed scans of western blots.

Figure 3f

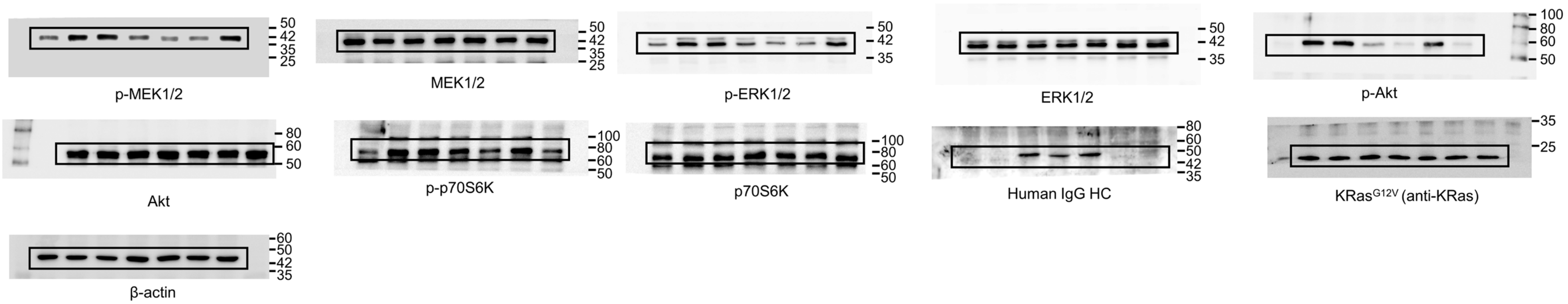

Figure 4e

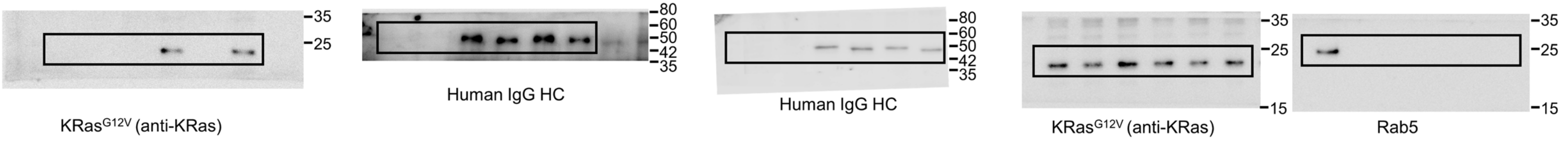

Supplementary Figure 12 Unprocessed scans of western blots. (Continued)

Supplementary Table 1 Binding constants for the interactions of RT11 and RT11-i with GppNHp-loaded active forms of Ras, determined by SPR.

Anti-bodies	Ras proteins	Kinetic parameters [a]		
		k_a ($M^{-1}s^{-1}$)	k_d (s^{-1})	K_D (M)
RT11	$KRas^{WT}$·GppNHp	$(2.77 \pm 0.72) \times 10^{5}$	$(3.30 \pm 0.15) \times 10^{-3}$	$(1.09 \pm 0.58) \times 10^{-8}$
	$KRas^{G12D}$·GppNHp	$(8.04 \pm 2.51) \times 10^{4}$	$(9.07 \pm 1.68) \times 10^{-4}$	$(1.11 \pm 0.29) \times 10^{-8}$
	$KRas^{G12V}$·GppNHp	$(9.11 \pm 1.17) \times 10^{4}$	$(1.41 \pm 0.49) \times 10^{-3}$	$(1.65 \pm 0.44) \times 10^{-8}$
	$KRas^{G13D}$·GppNHp	$(1.27 \pm 0.31) \times 10^{5}$	$(2.22 \pm 0.67) \times 10^{-3}$	$(1.55 \pm 0.84) \times 10^{-8}$
	$KRas^{Q61H}$·GppNHp	$(5.22 \pm 0.91) \times 10^{4}$	$(7.44 \pm 0.84) \times 10^{-4}$	$(1.58 \pm 0.51) \times 10^{-8}$
	$HRas^{WT}$·GppNHp	$(6.46 \pm 0.75) \times 10^{5}$	$(2.17 \pm 0.71) \times 10^{-3}$	$(3.62 \pm 1.32) \times 10^{-9}$
	$HRas^{G12V}$·GppNHp	$(2.55 \pm 1.02) \times 10^{5}$	$(1.41 \pm 0.52) \times 10^{-3}$	$(5.01 \pm 1.12) \times 10^{-9}$
	$NRas^{WT}$·GppNHp	$(3.19 \pm 1.11) \times 10^{4}$	$(3.55 \pm 0.48) \times 10^{-4}$	$(1.14 \pm 0.44) \times 10^{-8}$
	$NRas^{Q61R}$·GppNHp	$(3.31 \pm 0.66) \times 10^{4}$	$(3.68 \pm 0.65) \times 10^{-4}$	$(1.21 \pm 0.41) \times 10^{-8}$
RT11-i	$KRas^{WT}$·GppNHp	$(1.65 \pm 0.24) \times 10^{5}$	$(2.44 \pm 1.07) \times 10^{-3}$	$(1.44 \pm 1.51) \times 10^{-8}$
	$KRas^{G12D}$·GppNHp	$(3.33 \pm 0.55) \times 10^{4}$	$(6.84 \pm 2.64) \times 10^{-4}$	$(1.95 \pm 0.98) \times 10^{-8}$
	$KRas^{G12V}$·GppNHp	$(5.42 \pm 2.10) \times 10^{4}$	$(1.15 \pm 0.41) \times 10^{-3}$	$(2.16 \pm 0.72) \times 10^{-8}$
	$KRas^{G13D}$·GppNHp	$(8.01 \pm 1.72) \times 10^{4}$	$(1.85 \pm 0.60) \times 10^{-3}$	$(2.11 \pm 0.81) \times 10^{-8}$
	$KRas^{Q61H}$·GppNHp	$(2.67 \pm 0.77) \times 10^{4}$	$(4.95 \pm 0.44) \times 10^{-4}$	$(1.95 \pm 0.88) \times 10^{-8}$
	$HRas^{WT}$·GppNHp	$(4.41 \pm 1.60) \times 10^{5}$	$(1.62 \pm 0.78) \times 10^{-3}$	$(3.65 \pm 1.42) \times 10^{-9}$
	$HRas^{G12V}$·GppNHp	$(2.36 \pm 0.88) \times 10^{5}$	$(1.42 \pm 0.56) \times 10^{-3}$	$(5.58 \pm 0.51) \times 10^{-9}$
	$NRas^{WT}$·GppNHp	$(2.35 \pm 0.41) \times 10^{4}$	$(2.91 \pm 0.88) \times 10^{-4}$	$(1.11 \pm 0.89) \times 10^{-8}$
	$NRas^{Q61R}$·GppNHp	$(2.09 \pm 0.42) \times 10^{4}$	$(2.98 \pm 0.87) \times 10^{-4}$	$(1.39 \pm 0.83) \times 10^{-8}$

[a] Each value represents the mean ± s.d. of two independent experiments. In each experiment, at least five data sets were used to determine the kinetic constants. The dissociation (k_{off}) and association rate constants (k_{on}), and the dissociation equilibrium constant (K_D) values, were determined by the 1:1 Langmuir binding model using the BIAevaluation software provided by the manufacturer.

Supplementary Table 2 Quantitative assessment of cytosolic concentrations of RT11 and RT11-i in HeLa cells.

Parameters	Antibodies	Treated concentrations (μM)		
		0.1	0.5	1
Cytosolic amount (fmole)[a]	RT11	0.25 ± 0.12	1.51 ± 0.20	3.66 ± 0.20
	RT11-i	0.48 ± 0.03	2.41 ± 0.22	4.92 ± 0.29
Cytosolic concentration (nM)[b]	RT11	14 ± 6	80 ± 11	195 ± 11
	RT11-i	25.3 ± 2	135 ± 12	308 ± 18

[a] The cytosolic amount was measured by enhanced split-GFP complementation assay obtained by incubation of HeLa-SA-GFP1-10 cells with RT11-GFP11-SBP2 and RT11-i-GFP11-SBP2 antibodies, as described in the Methods and in our previous work[8].

[b] The cytosolic molar concentration was estimated by dividing the cytosolic amount by the cytosolic volume of HeLa cells and cell numbers in each well, as described in the Methods and in our previous work[8].

Supplementary Table 3 List of antibodies used in this study.

Antibody	Applications	Company	Catalog no.	Dilution
c-myc	Flow cytometry	Ig therapy	A03002	1: 250
Flag	Flow cytometry	Sigma-Aldrich	F3165	1: 250
FITC-conjugated anti-human IgG (Fc specific)	Flow cytometry	Sigma-Aldrich	F9512	1 : 200
Alexa Fluor 488-conjugated anti-mouse IgG	Flow cytometry	Invitrogen	A-11045	1 : 250
PE-conjugated anti-human integrin αvβ3	Flow cytometry	R&D systems,	FAB3050P,	1:100
PE-conjugated anti-human integrin αvβ5	Flow cytometry	R&D systems,	FAB2528P	1:100
HRP-conjugated anti-GST	ELISA	Sigma-Aldrich	A7340	1 : 4000
HRP-conjugated anti-His	ELISA	Sigma-Aldrich	A7058	1 : 2000
Anti-human IgG (Fab specific)	ELISA	Sigma-Aldrich	I5260	1 : 400
HRP-conjugated anti-human IgG	ELISA	Invitrogen	62-8420	1 : 8000
MEK1/2	Western blot	Cell signaling	9122	1 : 1000
phospho-MEK1/2	Western blot	Cell signaling	9121	1 : 1000
ERK1/2	Western blot	Cell signaling	4695	1 : 1000
phospho-ERK1/2	Western blot	Cell signaling	4377	1 : 1000
Akt	Western blot	Cell signaling	9272	1 : 1000
phospho-Akt	Western blot	Cell signaling	9271	1 : 1000
p70S6K	Western blot	Cell signaling	9202	1 : 1000
phospho-p70S6K	Western blot	Cell signaling	9205	1 : 1000
Rab5	Western blot	Cell signaling	3547	1 : 1000
HA	Western blot	Covance	MMS-101P	1 : 1000
KRas	Western blot	Santa Cruz	sc-30	1 : 500
bRaf	Western blot	Santa Cruz	sc-5284	1 : 500
cRaf	Western blot	Santa Cruz	sc-133	1 : 500
β-actin	Western blot	Santa Cruz	sc-69879	1 : 1000
HRP-conjugated anti-mouse IgG	Western blot	Santa Cruz	sc-2031	1 : 4000
HRP-conjugated anti-rabbit IgG	Western blot	Santa Cruz	sc-2004	1 : 4000
HRP-conjugated anti-goat IgG	Western blot	Santa Cruz	sc-2033	1 : 4000
Anti-human IgG (Fc specific)	Western blot	Invitrogen	31125	1 : 1000
Ras	Immunofluorescence Immunohistochemistry	Abcam	ab108602	1 : 100
Alexa Fluor 488-conjugated anti-human IgG	Immunofluorescence Immunohistochemistry	Invitrogen	A-11013	1 : 500
TRITC-conjugated anti-rabbit IgG	Immunofluorescence Immunohistochemistry	Sigma-Aldrich	T6778	1 : 250
phospho-ERK1/2	Immunohistochemistry	Abcam	ab9272	1 : 100
Ki-67	Immunohistochemistry	Abcam	ab15580	1 : 100
phospho-Akt	Immunohistochemistry	Cell signaling	9271	1 : 100
phospho-STAT3 (T705)	Immunohistochemistry	Cell signaling	9145	1 : 100
phospho-STAT3 (S727)	Immunohistochemistry	Cell signaling	9134	1 : 100
FITC-conjugated anti-rabbit IgG	Immunohistochemistry	Chemicon	AP132F	1 : 250

Supplementary References

1. Sun, Q. *et al.* Discovery of small molecules that bind to K-Ras and inhibit Sos-mediated activation. *Angew. Chem. Int. Ed. Engl.* **51**, 6140-6143 (2012).
2. Scheidig, A. J., Burmester, C. & Goody, R. S. The pre-hydrolysis state of p21(ras) in complex with GTP: new insights into the role of water molecules in the GTP hydrolysis reaction of ras-like proteins. *Structure* **7**, 1311-1324 (1999).
3. Ye, M. *et al.* Crystal structure of M-Ras reveals a GTP-bound "off" state conformation of Ras family small GTPases. *J. Biol. Chem.* **280**, 31267-31275 (2005).
4. Cash, J. N., Davis, E. M. & Tesmer, J. J. Structural and Biochemical Characterization of the Catalytic Core of the Metastatic Factor P-Rex1 and Its Regulation by PtdIns(3,4,5)P3. *Structure* **24**, 730-740 (2016).
5. Karnoub, A. E. & Weinberg, R. A. Ras oncogenes: split personalities. *Nat. Rev. Mol. Cell Biol.* **9**, 517-531 (2008).
6. Matsumoto, K. *et al.* Critical roles of interactions among switch I-preceding residues and between switch II and its neighboring alpha-helix in conformational dynamics of the GTP-bound Ras family small GTPases. *J. Biol. Chem.* **286**, 15403-15412 (2011).
7. Hunter, J. C., Manandhar, A., Carrasco, M. A., Gurbani, D., Gondi, S. & Westover, K. D. Biochemical and Structural Analysis of Common Cancer-Associated KRAS Mutations. *Mol. Cancer Res.* **13**, 1325-1335 (2015).
8. Kim, J. S. *et al.* Quantitative assessment of cellular uptake and cytosolic access of antibody in living cells by an enhanced split GFP complementation assay. *Biochem. Biophys. Res. Commun.* **467**, 771-777 (2015).
